# A Method for Simultaneous Evaluation of Muscular and Neural Prepulse Inhibition

**DOI:** 10.3389/fnins.2018.00654

**Published:** 2018-09-26

**Authors:** Rodrigo San-Martin, Maria Inês Zimiani, Claudemiro Noya, Milton Augusto Vendramini Ávila, Rosana Shuhama, Cristina Marta Del-Ben, Paulo Rossi Menezes, Francisco José Fraga, Cristiane Salum

**Affiliations:** ^1^Center for Mathematics, Computation and Cognition, Universidade Federal do ABC, São Bernardo do Campo, Brazil; ^2^Engineering, Modeling and Applied Social Sciences Center, Universidade Federal do ABC, Santo André, Brazil; ^3^Ribeirão Preto Medical School, Universidade de São Paulo, Ribeirão Preto, Brazil; ^4^Department of Preventive Medicine, Faculdade de Medicina, Universidade de São Paulo, São Paulo, Brazil; ^5^Population Mental Health Research Center, Universidade de São Paulo, São Paulo, Brazil

**Keywords:** electroencephalogram (EEG), event-related potentials (ERP), fully automated artifact removal, independent component analysis (ICA), prepulse inhibition (PPI), sensory gating, sensorimotor gating

## Abstract

Prepulse inhibition (PPI) test has been widely used to evaluate sensorimotor gating. In humans, deficits in this mechanism are measured through the orbicularis muscle response using electromyography (EMG). Although this mechanism can be modulated by several brain structures and is impaired in some pathologies as schizophrenia and bipolar disorder, neural PPI evaluation is rarely performed in humans. Since eye blinks are a consequence of PPI stimulation, they strongly contaminate the electroencephalogram (EEG) signal. This paper describes a method to reduce muscular artifacts and enable neural PPI assessment through EEG in parallel to muscular PPI evaluation using EMG. Both types of signal were simultaneously recorded in 22 healthy subjects. PPI was evaluated by the acoustical startle response with EMG and by the P2-N1 event-related potential (ERP) using EEG in Fz, Cz, and Pz electrodes. In order to remove EEG artifacts, Independent Component Analysis (ICA) was performed using two methods. Firstly, visual inspection discarded components containing artifact characteristics as ocular and tonic muscle artifacts. The second method used visual inspection as gold standard to validate parameters in an automated component selection using the SASICA algorithm. As an outcome, EEG artifacts were effectively removed and equivalent neural PPI evaluation performance was obtained using both methods, with subjects exhibiting consistent neural as well as muscular PPI. This novel method improves PPI test, enabling neural gating mechanisms assessment within the latency of 100–200 ms, which is not evaluated by other sensory gating tests as P50 and mismatch negativity.

## Introduction

The central nervous system has a protective filtering mechanism, named sensory gating (Boutros et al., 2004), which acts reducing low salience sensorial inputs and preventing superior cortical systems from an overload of information (Kumari et al., 2012). This feature may be evaluated with the prepulse inhibition (PPI) test of acoustic startle reflex (ASR), a phenomenon described by the reduced muscular response elicited by a high intensity stimulus, pulse (P), when it is preceded in a few milliseconds (30–300 ms) by a low intensity stimulus, prepulse (PP) (Koch, 1999).

In order to assess PPI in humans, electromyographic (EMG) activity of the orbicularis muscle, which controls eyelid closure and opening, is recorded by electrodes placed below the eye (Braff et al., 1992; Blumenthal et al., 2005). Although brain processes involved during sensory gating have a central role in PPI (Swerdlow et al., 2016), scientific research literature lacks investigation of neural mechanisms underlying this phenomenon in humans.

So far, few studies have developed this analysis, mostly using electroencephalography (EEG) (Abduljawad et al., 2001; Kedzior et al., 2007; De Pascalis et al., 2013; Sommer et al., 2016). One of the reasons is that the required stimulation to elicit startle and measure PPI through EMG evokes muscular artifacts that contaminate the EEG signal, thus hindering simultaneous neural PPI evaluation. Herein we propose a method to attenuate those muscular artifacts, enabling neural PPI evaluation through EEG simultaneously to muscular PPI assessment with EMG.

## Materials and Methods

### Participants and Procedure

This study was approved by UFABC Research Ethics Committee. All participants gave written informed consent in accordance with the Declaration of Helsinki. Participants were 22 healthy subjects; (15 males, 7 females), absent of neurologic or psychiatric disorder, already enrolled in the “Schizophrenia and other psychosis Translational Research: Environment and Molecular Biology” (STREAM) project. Mean ± standard deviation age = 26.00 ± 5.36, education years = 12.4 ± 2.66, and Brief Psychiatric Rating Scale (BPRS) = 0.24 ± 2.26.

### Experimental Design

Subjects sat in a comfortable chair and were instructed they would hear binaural sounds through headphones. Following an acclimation period, two identical blocks of PPI stimuli were presented with 3 min inter-blocks-interval. Blocks consisted of pseudorandomly presented P and PP+P (30, 60, and 120 ms interval, henceforward P30, P60, and P120) stimuli, 20 of each. Additionally, five P were presented before block 1 and after block 2. PP and P were white noises of 85 and 115 dB, with 20 and 40 ms duration, respectively. During acclimation and PPI blocks, 70dB background white noise was present.

### Equipment

Electroencephalogram (EEG) and Electromyography (EMG). were recorded simultaneously at 512 Hz sampling rate using a dry-active electrode cap (BrainVision, actiCAP Xpress, BrainProducts-Germany) with 11 scalp channels following the standard 10–20 International System, two channels for EMG, one channel for EOG (Electrooculogram), and one for additional reference. The ASR was recorded through EMG using electrodes placed 2 cm below the pupil (EMG1) and 2 cm below right eyes outer end (EMG2). Primary reference was attached to the right earlobe with the additional reference electrode placed on the left earlobe.

### Signal Processing

MATLAB software running the EEGLAB package (Delorme and Makeig, 2004) and SASICA plug-in (Chaumon et al., 2015) were used in signal processing. Signal was referenced to the average of right and left earlobes electrodes and filtering was performed in “zero phase” mode using fourth-order Butterworth filters with highpass-(0.25 Hz) and notch-filter at 60 Hz to eliminate power grid frequency and its harmonics.

#### EMG Processing

EMG1 and EMG2 formed EMG. Bandpass filter (24–200 Hz) was applied in order to reduce low-frequency artifacts and reinforce orbicularis muscle activity in the signal (Blumenthal et al., 2005). EMG (absolute) envelope was then obtained with rectification followed by low-pass filtering (15.9 Hz). Afterwards, the signal was divided into 600 ms segments, from −300 to +300 ms in relation to P stimulus (which occurs at 0 ms), and the pre-stimulus average baseline was calculated using the 50 ms before the auditory stimulus. For each trial, the ASR was considered as the maximum blink amplitude in the 20–120 ms interval.

The mean ASR for each participant and experimental condition was calculated as the average of all trials belonging to that subject and condition. Participants were classified as non-responders and excluded from the final sample when the mean ASR to P was less than 20 digital units (0.0488^∗^20 μV). Trials were discarded if maximum value was three times above the mean standard deviation for that specific stimulus and if the trials baseline was three times above the standard deviation for the baseline of the subject. Six subjects were classified as non-responders; sixteen remaining underwent PPI analysis.

#### EEG Processing

In order to obtain event-related potentials (ERPs), EEG signal was partitioned in −1 to 1 s epochs in relation to P stimulus and the baseline was calculated according to average activity in the −650 to −150 ms interval. Then, all epochs were low-pass filtered (40 Hz).

To completely remove ocular (blinking and saccades) and other muscular (transient and ongoing) artifacts from the neural signal, Independent Component Analysis (ICA) was performed using the INFOMAX algorithm implemented in the EEGLAB toolbox (Delorme et al., 2007). For artifactual components removal, two separate paths were followed. In the first one, termed Visual ICA (semi-automated method), the independent components (ICs) were visually inspected and removed (by an experienced analyst), whenever they had characteristics of ocular and/or muscular artifacts (Chaumon et al., 2015). The second path, named SASICA (fully automated method), used SASICA algorithm with parameters adjusted based on Visual ICA method (“gold standard”) until the final result was as close as possible to the one obtained by the semi-automated method. Parameters to classify artifactual ICs are Autocorrelation, with threshold (r) auto and Lag 20; Focal components, with threshold (z) 3.5; Focal trial activity, with threshold (z) 10; Signal to noise ratio POI (min max ms) 0 to Inf, BL (min max ms) −Inf to 0 and threshold ratio 1; Combination with EOG, EMG1, and EMG2 threshold (r) 0.2; ADJUST and FASTER enabled with blink channels.

For each subject, epochs were averaged separately for each of the four types of auditory stimuli (P, PP30+P, PP60+P, and PP120+P). For all scalp channels, ERP N100 (or N1) was identified as the most negative peak in the 60–165 ms interval and ERP P200 (or P2) as the most positive peak in the 165–275 ms interval.

#### PPI

Both muscular (calculated on the ASR) and neural (calculated on the ERP) PPI were computed according to the formula: *%PPI* = 100^∗^[1 − (*P − PP*)]/*P*.

### Statistics

ERP analysis was restricted to the three central electrodes (Fz, Cz, and Pz) for P2-N1 ERP (Abduljawad et al., 2001). Data distribution was evaluated for normality and Repeated Measures (RM)-ANOVA was employed for normally distributed data. When data did not pass normality test, the non-parametric Friedman test was applied. Subsequently, in case of significant differences, pairwise comparisons *post hoc* analyses were performed by Tukey test for parametric data and by Wilcoxon tests with Bonferroni adjustment for non-parametric data. Pairwise comparisons were applied for amplitude (P vs. P30 or P60 or P120) and percentage (PPI30 vs. PPI60, PPI30 vs. PPI120, and PPI60 vs. PPI120) conditions. Kendall correlation analyses for %PPI between Fz, Cz, and Pz and EMG were also performed.

## Results

PPI amplitude and percentage comparison between the different intervals were calculated for muscular response through ASR and for neural response through P2-N1 ERP complex. Descriptive statistics, corresponding *p*-values for main effects and pairwise comparisons are displayed in **Table 1**.

**Table 1:** Percentage of Prepulse Inhibition and Amplitude values evaluated through ASR (muscular PPI) and P2-N1 ERP complex (neural PPI).

Startle and event related potentials	ASR	P2-N1 at Fz	P2-N1 at Cz	P2-N1 at Pz
**Percentage values**				
%PPI30 (Mean ± SE / Median)	37.37 ± 5.80 / 40.43	37.96 ± 9.25 / 44.81	38.85 ± 4.71 / 44.88	45.41 ± 4.69 / 42.16
%PPI60 (Mean ± SE / Median)	56.71 ± 4.95 / 60.78	53.16 ± 10.96 / 67.17	54.12 ± 10.65 / 62.67	56.42 ± 5.14 / 59.42
%PPI120 (Mean ± SE / Median)	33.84 ± 7.54 / 38.39	40.57 ± 10.32 / 49.96	38.31 ± 9.86 / 48.80	41.63 ± 4.55 / 36.96
Group effect (p)	0.0009^a^	0.0052^b^	0.0006^b^	0.0720^a^
Multiple comparisons (p)				
PPI30 × PPI60	0.0120	0.0064	0.0229	-
PPI30 × PPI120	n.s	n.s.	n.s.	-
PPI60 × PPI120	0.0030	0.0049	0.004	-
**Amplitude values (**μV**)**				
P (Mean ± SE / Median)	3.87 ± 0.96 / 2.16	30.97 ± 3.64 / 32.09	31.95 ± 3.66 / 31.64	23.31 ± 2.07 / 22.16
P30 (Mean ± SE / Median)	2.31 ± 0.63 / 1.46	16.23 ± 1.81 / 15.16	18.80 ± 2.31 / 17.45	11.98 ± 1.22 / 11.87
P60 (Mean ± SE / Median)	1.34 ± 0.25 / 1.13	11.45 ± 1.39 / 10.11	12.30 ± 1.80 / 11.23	10.70 ± 2.38 / 8.10
P120 (Mean ± SE / Median)	2.37 ± 0.55 / 2.37	15.42 ± 1.58 / 12.60	18.03 ± 2.39 / 14.72	13.83 ± 2.12 / 10.54
Group effect (p)	<0.0001^b^	<0.0001^a^	<0.0001^b^	<0.0001^b^
Multiple comparisons (p)				
P × P30	0.0004	<0.0001	0.0002	<0.0001
P × P60	0.0002	<0.0001	0.0003	<0.0001
P × P120	0.0050	<0.0001	0.0096	0.0014

*^a^Repeated Measures ANOVA for %PPI or Amplitude differences between P and P+PP intervals. ^b^ Friedman Test for %PPI or Amplitude differences between P and P+PP intervals.*

For muscular PPI (ASR), Friedman test revealed that ASR differed significantly between P, P30, P60, and P120 conditions [χ2(3) = 25.125, *p* < 0.0001]. *Post hoc* analysis revealed that P30, P60, and P120 were significantly reduced when compared to P. RM-ANOVA showed that mean %PPI differed significantly between PPI30, PPI60, and PPI120 [*F*(2,30) = 8.987, *p* < 0.0008]. Tukey *post hoc* tests revealed that PPI30 and PPI120 were reduced when compared to PPI60.

At Fz, Cz, and Pz scalp location, RM-ANOVA and Friedman test showed that P2-N1 Amplitude differed significantly between P, P30, P60, and P120 [Fz: *F*(3,45) = 30.099, *p* < 0.0001; Cz: χ2(3) = 31.575, *p* < 0.0001; Pz: χ2(3) = 34.500, *p* < 0.0001]. *Post hoc* analysis for the three electrodes revealed that P30, P60, and P120 amplitude were significantly reduced when compared to P. Friedman test revealed that Fz and Cz P2-N1 %PPI differed significantly between PPI30, PPI60, and PP120 [Fz: χ2(2) = 10.508, *p* = 0.0052; Cz: χ2(2) = 14.952, *p* = 0.0006]. *Post hoc* analysis revealed that Fz and Cz PPI120 and PPI30 were reduced in comparison to PPI60.

Kendall correlation analysis for each %PPI condition (PPI30, PPI60, and PPI120) between EMG and ERPs for each electrode (Fz, Cz, and Pz) revealed no correlation among neural and muscular PPI.

**FIGURE 1:**
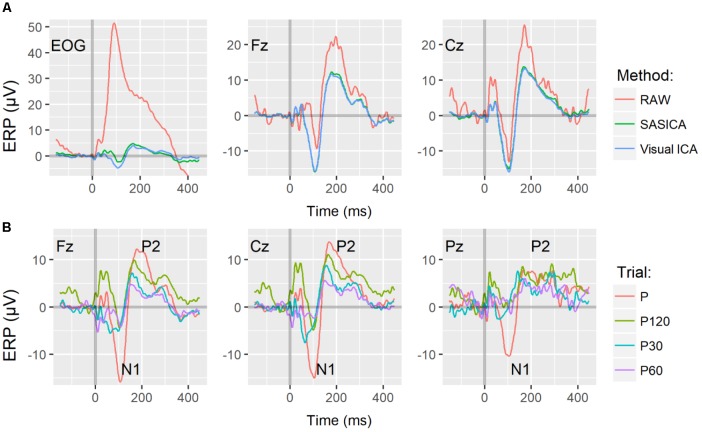
**(A)** Pulse Alone (P) Grand Averages (*N* = 16) of ERPs at electrodes EOG, Fz, and Cz corrected by visual inspection of ICs (Visual ICA, in blue) and by the fully automated method (SASICA, in green). In red, the same grand averages performed over raw data ERP (RAW) displayed for comparison. **(B)** Event Related Potentials (ERP) filtered with the automated method displayed for P (red) and PP+P with ISI 30, 60, and 120 ms, P30 (green), P60 (blue), and P120 (purple), respectively, for Fz, Cz, and Pz electrodes. For **(A,B)**, time 0 (s) indicates the onset of the 115 dB SPL auditory pulse.

ERP P Grand averages at electrodes EOG, Fz, and Cz are shown in **Figure 1A**. For comparison, the same grand averages performed over raw data (RAW), i.e., without artifact correction procedure, are also displayed. Both, semi and fully automated artifact correction methods removed artifacts, as shown by the ERP at EOG. In **Figure 1B**, automated artifact corrected ERPs of P and the three PP+P conditions are displayed for Fz, Cz, and Pz electrodes, indicating amplitude reduction in P2 and N1 when P is preceded by PPs, i.e., neural PPI.

## Discussion

Here we demonstrate an efficient method to attenuate artifacts intrinsically produced by PPI stimulation eye blinks, enabling neural PPI (ERP through EEG) evaluation simultaneously with muscular PPI (ASR through EMG). To the best of our knowledge, this is the first study addressing the usage of automatic (or even semi-automatic) artifact removal tools to enhance the neural PPI signal.

In line with previous study (Kedzior et al., 2007), no correlation was identified between muscular and neural PPI. This finding is expected, as the ASR is a 20–120 ms latency motor response, elicited in the lower brainstem with sensorimotor gating mechanisms modulating the caudal pontine reticular nucleus (Koch, 1999), while the N1 and P2 ERPs are neural activity outcomes providing information on neural processing stages. Another singular aspect of neural PPI, is that it evaluates neural gating phenomenon in the time window of 100–200 ms. This latency is not investigated by other sensory gating paradigms, as the paired-click P50 test assesses inhibitory mechanisms 550 ms after first or 50 ms after the second clicks, while the deviant stimuli MMN paradigm evaluates the latency of 150–350 ms. Thus, neural PPI may offer novel insights onto gating mechanisms in humans.

Due to technological limitations, past studies analyzing EEG-PPI did not mention artifact removal (Abduljawad et al., 2001) or removed epochs with blinks unsynchronized to auditory stimulation, considering synchronized blinks as non-artifactual components (Kedzior et al., 2007). More recently, other authors used statistical thresholding to remove artifactual epochs (De Pascalis et al., 2013; Sommer et al., 2016). The problem of applying statistical thresholding to PPI studies relies on the fact that trials with stronger eye blinks reinforce ASR-PPI, but, contradictorily, are removed from neural PPI, decreasing the value of parallel comparison for these effects, since they are not evaluated over the same trials. Our method might be a more suitable option to deal with eye-related artifacts because it maintains similar trials for both muscular and neural PPI assessment.

Our findings also demonstrate that artifactual components strongly contaminate raw PPI neural signal. The difference between raw to ICA-cleaned signal is higher in frontal electrodes, as they are closer to eye-related muscular regions than central and parietal electrodes. Remarkably, artifact removal allowed even the EOG signal to reveal reduced (as compared to scalp electrodes) but clear N1 and P2 ERPs (**Figure 1A**). This graphical clue might indicate that both applied methods were effective for muscular components removal.

The proposed method for neural PPI evaluation seems to be quite promising, but some methodological limitations must be considered. First, further studies with larger EEG-PPI databases are required to further validate the method. Second, although artifactual components identification using ICA is straightforward for blinks, saccades, muscular and rare events components, some components are tricky and present artifactual and neural signal characteristics at the same time, which is an obstacle for analyst’s decisions exact replication in those cases. This is the main reason that in our method we proposed the use of an automated process, in order to standardize the analysis among different laboratories. Even though in our method we used the Visual ICA as gold standard, the selection process in the automated algorithm avoids inter-subject selection bias and in case of future studies also reduces possible inter-group selection bias. Third, EMG-PPI was evaluated on a large amount of trials for comparison purposes with EEG-PPI. Future works may consider reduction in the number of trials for the former. Fourth, evaluation over a wider range of electrodes should be considered, as our study was limited to Fz, Cz, and Pz. Lastly, EEG-PPI evaluation in subjects that typically present deficits in EMG-PPI, such as schizophrenia patients (Swerdlow et al., 2017), is highly encouraged in order to assess if neural PPI analysis offers new insights in sensory gating investigation complementarily to muscular PPI.

In this study, we described a method to evaluate muscular and neural PPI simultaneously. Further research is needed to validate neural PPI with a higher number of healthy subjects as well as patient groups before more generalized conclusions can be drawn.

## Author Contributions

PM, CS, and CD-B conceived the idea. RS-M, MZ, CN, FF, and CS developed the methodological and analytical strategies. MZ, MÁ, RS, CD-B, and CS recruited and acquired the data. RS-M, CN, FJ, and CS analyzed the data. FJ and CN semi-automated the ICA. RS-M and FJ automated the ICA. RS-M drafted the manuscript. FJ and CS reviewed the manuscript. RS-M, MZ, MÁ, RS, CD-B, PM, FF, and CS critically reviewed the manuscript. All authors read and approved the final manuscript.

## Conflict of Interest Statement

The authors declare that the research was conducted in the absence of any commercial or financial relationships that could be construed as a potential conflict of interest.
